# Long-range dispersal moved *Francisella tularensis* into Western Europe from the East

**DOI:** 10.1099/mgen.0.000100

**Published:** 2016-12-12

**Authors:** Chinmay Dwibedi, Dawn Birdsell, Adrian Lärkeryd, Kerstin Myrtennäs, Caroline Öhrman, Elin Nilsson, Edvin Karlsson, Christian Hochhalter, Andrew Rivera, Sara Maltinsky, Brittany Bayer, Paul Keim, Holger C. Scholz, Herbert Tomaso, Matthias Wittwer, Christian Beuret, Nadia Schuerch, Paola Pilo, Marta Hernández Pérez, David Rodriguez-Lazaro, Raquel Escudero, Pedro Anda, Mats Forsman, David M. Wagner, Pär Larsson, Anders Johansson

**Affiliations:** ^1^​ Department of Clinical Microbiology and the Laboratory for Molecular Infection Medicine Sweden, Umeå University, Umeå, Sweden; ^2^​ Division of CBRN Security and Defence, Swedish Defense Research Agency, Umeå, Sweden; ^3^​ Center for Microbial Genetics and Genomics, Northern Arizona University, Flagstaff AZ, USA; ^4^​ Translational Genomics Research Institute North, Flagstaff AZ, USA; ^5^​ Bundeswehr Institute of Microbiology, Munich, Germany; ^6^​ Institute of Bacterial Infections and Zoonoses Friedrich-Loeffler, Institut Federal Research Institute for Animal Health, Jena, Germany; ^7^​ Biology Division, Spietz Laboratory, Federal Office for Civil Protection, Spietz, Switzerland; ^8^​ Institute of Veterinary Bacteriology, University of Bern, Bern, Switzerland; ^9^​ Laboratory of Molecular Biology and Microbiology, Instituto Tecnológico Agrario de Castilla y León, Valladolid, Spain; ^10^​ Departamento de Ingeniería Agrícola y Forestal, Universidad de Valladolid, Palencia, Spain; ^11^​ Department of Biotechnology and Food science, Universidad de Burgos, Burgos, Spain; ^12^​ Centro Nacional de Microbiología, Instituto de Salud Carlos III, Madrid, Spain

**Keywords:** epidemiology, disease transmission, human, population genetics, Francisella tularensis, genetic variation

## Abstract

For many infections transmitting to humans from reservoirs in nature, disease dispersal patterns over space and time are largely unknown. Here, a reversed genomics approach helped us understand disease dispersal and yielded insight into evolution and biological properties of *Francisella tularensis*, the bacterium causing tularemia. We whole-genome sequenced 67 strains and characterized by single-nucleotide polymorphism assays 138 strains, collected from individuals infected 1947-2012 across Western Europe. We used the data for phylogenetic, population genetic and geographical network analyses. All strains (*n*=205) belonged to a monophyletic population of recent ancestry not found outside Western Europe. Most strains (*n*=195) throughout the study area were assigned to a star-like phylogenetic pattern indicating that colonization of Western Europe occurred via clonal expansion. In the East of the study area, strains were more diverse, consistent with a founder population spreading from east to west. The relationship of genetic and geographic distance within the *F. tularensis* population was complex and indicated multiple long-distance dispersal events. Mutation rate estimates based on year of isolation indicated null rates; in outbreak hotspots only, there was a rate of 0.4 mutations/genome/year. Patterns of nucleotide substitution showed marked AT mutational bias suggestive of genetic drift. These results demonstrate that tularemia has moved from east to west in Europe and that *F. tularensis* has a biology characterized by long-range geographical dispersal events and mostly slow, but variable, replication rates. The results indicate that mutation-driven evolution, a resting survival phase, genetic drift and long-distance geographical dispersal events have interacted to generate genetic diversity within this species.

## Data Summary

This study uses whole-genome sequence data of *F. tularensis* samples and connected metadata, which are available through the information given in Table S1 (available in the online Supplementary Material) including the GenBank accession numbers. The single-nucleotide polymorphisms and the canSNPer software used for the canSNP analysis are available at https://github.com/adrlar/CanSNPer.

## Impact Statement

In this work, we used genome data to understand biological properties and geographical spread of the bacterium *Francisella. tularensis,* which causes the disease tularemia. Humans may contract tularemia from infected mammals, ticks or mosquitoes or from environmental dust, but it is unclear where the bacterium survives between infections. By mapping the genomes of *F. tularensis* strains from many infected individuals across Western Europe, we found that tularemia has moved from east to west in Europe. Unexpectedly, we observed a movement pattern of big jumps across the continent. Our study advances the research field by showing that *F. tularensis* has a mechanism for long-distance transport. We additionally found more evidence that *F. tularensis* spends much of the time in a resting survival phase between infection episodes. More generally, this work demonstrates the value of analysing microbial genome data at large scales for learning more about an infectious organism´s biology and for interpreting epidemiological patterns of infectious diseases that currently are poorly understood.

## Introduction

Geographical dispersal of microbes causing disease can be difficult to study by genetic approaches, mainly because dispersal of microbes may be rapid in relation to the rate of mutation and genetic diversification is often characterized by horizontal gene transfer events that can quickly obscure phylogenetic signatures of dispersal. It remains uncertain if barriers to geographical dispersal exist for microbes which could influence the genetic diversity of populations and would be analogous to those observed among plants and animals (Finlay, 2002; Nemergut *et al.*, 2013). With the advent of large-scale genetic population approaches for microbes more knowledge is accumulating; results from basal studies in saline environments, experimental systems and soils indicate that spatial distance may contribute to microbial genetic diversity patterns (Low-Décarie *et al.*, 2015; Ramette & Tiedje, 2007; Wang *et al.*, 2015). These approaches may additionally provide novel insights into an organism´s biology (Vellend *et al.*, 2014).

*Francisella tularensis,* a facultative intracellular bacterium causing the disease tularemia, is best known as a potential agent of bioterrorism due to its high virulence, low infectious dose and ease of spread by aerosol; it historically was stockpiled as a biological weapon (Dennis *et al.*, 2001). Natural disease outbreaks present an opportunity to investigate microbial population diversity and geographical dispersal of *F. tularensis,* which is a bacterium with little genetic variation (Johansson & Petersen, 2010). Dispersal can be studied by investigating *F. tularensis* isolated from various geographic locations from diseased humans, other mammals, or transmitting arthropod vectors. There are two subspecies of *F. tularensis* important with respect to infection in mammals of which only the less aggressive *F. tularensis* subspecies *holarctica* exists in Europe (Dennis *et al.*, 2001).

Tularemia has only recently been reported from Western Europe and it appears that a single genetic subpopulation of *F. tularensis* is specific to this region (Dempsey *et al.*, 2007; Gyuranecz *et al.*, 2012; Pilo *et al.*, 2009; Vogler *et al.*, 2009). Seasonal disease outbreaks of tularemia were first reported in central Europe in the late 1930s around Marchfeld, north of Vienna, and continued to appear intermittently in Austria, Czechoslovakia, Poland and Eastern Germany throughout the next decade (Jusatz, 1952b). In the early 1950s independent outbreaks were documented in Western Germany and France (Correspondent, 1947; Gelman, 1961; Jusatz, 1952b). Italy reported its first tularemia outbreak only in 1964 and Spain as recently as 1997 (Gutiérrez *et al.*, 2003; Instituto de Salud Carlos III, 1997; Rinaldi *et al.*, 1964).

We applied whole-genome and canonical single-nucleotide polymorphism (canSNP) analysis to a comprehensive set of *F. tularensis* samples from Western Europe to characterize microbial genetic diversity through the lens of the four classical processes of population genetics: selection, genetic drift, mutation and dispersal. Our findings demonstrate that tularemia has moved east to west in Europe in big jumps.

## Methods

### Study location and data collection.

A total of 205 *F. tularensis* subspecies *holarctica* strains were collected from countries in continental Western Europe, including: Belgium, Germany, France, Netherlands, Italy, Spain and Switzerland. These strains were isolated over 65 years (1947–2012) from infected humans, infected mammals in zoos, arthropod vectors, including ticks, and free-ranging wild animals. Thus, they represent a very diverse set of hosts and vectors over large spatial and temporal scales.

### Genome sequencing.

Whole-genome sequences were used to identify SNPs in 67 *F. tularensis* samples from Western Europe. A set of 62 whole-genome sequences were generated for this study using Illumina sequencing platforms (Illumina) and five were retrieved from the public domain (see Table S1). The sequencing instruments used were HiSeq 2000 (SciLifeLabs, Uppsala, Sweden and Spietz laboratory, Spietz, Switzerland), GA IIx [Translational Genomics Research Institute (TGen), Flagstaff, Arizona], and MiSeq [Swedish Defence Research Agency (FOI), Umeå, Sweden; Northern Arizona University and TGen, Flagstaff, USA]. Steps of DNA preparation, library construction, and genome sequencing were done according to the manufacturer’s instructions. Library preparations were performed using TruSeq kits (Illumina), Nextera XT kits (Illumina), or KAPA library preparation kits (KAPA Biosystems). The KAPA kits were used with Illumina sequencing per a modified protocol including the incorporation of customized 8 bp tags for multiplexing (Kozarewa & Turner, 2011), with the adapters and oligos purchased from IDT (Integrated DNA Technologies).

### CanSNP assays.

Data from comparisons of the whole genomes were used to construct 20 new canSNP assays for the characterization of *F. tularensis* strain samples following previously published guidelines (Birdsell *et al.*, 2012). In addition, assays described in previous studies were used (Svensson *et al.*, 2009; Vogler *et al.*, 2009) to assign each sample to a phylogenetic subpopulation defined by canSNPs (see Tables S1, S2 and S3 for more information on samples and canSNP assays including primer concentrations and PCR conditions).

### Genomic assembly and alignment.

**Fig. 1.**
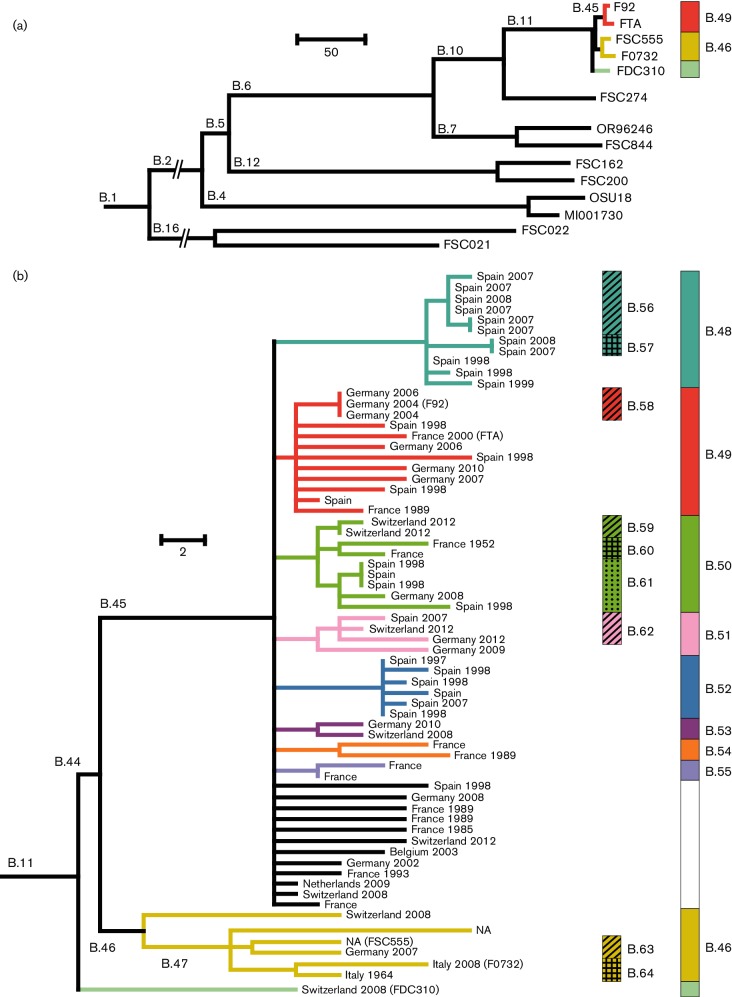
Whole genome neighbor-joining phylogenetic trees representing relationships among *F. tularensis* strains. (a) shows the relationships of 67 strains from Western Europe (Branch B.11) relative to the known global diversity within *F. tularensis* subsp. *holarctica*. (b) shows detailed relationships among strains from Western Europe. Country of origin and year of isolation are indicated at the branch tips, with colors representing different phylogenetic clades.

The *F. tularensis* genome sequences from the study region were assembled using ABySS 1.5.2 (Simpson *et al.*, 2009) and compared with a global database of more than 600 *F. tularensis* genomes maintained at the Swedish Defense Research Agency, Umeå, Sweden. All *F. tularensis* genomes in our database that were found to differ by less than 10 SNPs from any genome in the study region, and nine additional public genome sequences, were used to generate a global diversity tree (see Fig. 1a). Genome alignments were generated using a stepwise procedure: (1) each sequence was aligned with the *F. tularensis* strain FSC200 genome (NC 009749) to generate a nucleotide position reference. (2) All genomes were merged into a single alignment that was visually reviewed for misalignments around gaps. (3) Five nucleotides upstream and five downstream of an alignment gap were excluded to remove uncertain SNPs because read alignment in these regions is error-prone.

### Phylogeny and genetic diversity.

The software mega v. 5.13 (Tamura *et al.*, 2011) was used for the calculation of genomic distances and for phylogenetic analysis of genomic data, employing the number of differences-model and the neighbor-joining algorithm. The mean nucleotide diversity (Pi) per country was calculated using mega for countries with more than five genomes. Pi for a comparison of the East and the West part of the study region was estimated using DnaSP 5.10.01 (Librado & Rozas, 2009). Using the genome-based phylogeny and the strategically selected canSNP assays representing the branches of this phylogeny, strains with canSNP data were assigned to a node of the tree. The canSNP approach was highly accurate for node assignments but did not expose potential new genetic diversity as compared with the genomes used for reconstructing the whole-genome tree (Alland *et al.*, 2003; Pearson *et al.*, 2004).

### Phylogeographic analysis.

Each sample was assigned to a whole-genome phylogenetic clade or to a canSNP group, mapped to geographical coordinates using Google Maps, and geographical clustering was generated by Marker Clusterer (https://github.com/googlemaps/js-marker-clusterer). The genetic network analysis was manually performed by connecting locations with identical *F. tularensis* canSNP genotypes. The ties connecting two locations were drawn to reflect the number of shared unique genotypes. The network was manually drawn as an arc diagram.

### Analysis of genetic to geographic distance.

Genetic clades of the whole-genome phylogeny containing more than five genomes were identified and used to analyze the relationship between genetic and geographic distances. A genetic distance matrix for all pairs within a clade was created using the SNP distance between strains, and a corresponding geographic distance matrix was created using the fossil package in R 2.10 (Vavrek, 2011).

### Historic and contemporary endemic regions.

Data on the spatial distribution of tularemia in Europe 1926–1955 were retrieved from publications by Jusatz (Jusatz, 1952a, b; 1955; 1961), and compared with the 1947–2012 data of this study. Comparison with the historic disease distribution was made by plotting instances of more than five strains located nearby as a cluster on a map, and by showing all strains located outside the historic disease distribution.

### Mutation rate analysis.

Mutation rate estimates were made using the software BEAST 1.8.1 (Drummond et al, 2012) with 100 million iterations, out of which 10 million were used as burn-in. The lognormal relaxed clock model and the GTR without site heterogeneity substitution model was selected. The full 67 genome dataset and the set of genomes from two outbreaks in Spain (Ariza-Miguel et al, 2014) were utilized in separate analyses.

### Nucleotide sequence accession numbers.

Whole-genome sequence data have been deposited at in GenBank. Accession numbers of sequence data and metadata for each sample are available in Table S1.

## Results

### Phylogeny for *F. tularensis* in Western Europe

Using a whole-genome assembly approach and SNP discovery, 67 *F. tularensis* strains from Western Europe were found to form a tightly clustered population distinct from all other worldwide *F. tularensis* subsp. *holarctica* genome sequences selected to represent the currently known genetic diversity of the subspecies (Fig. 1a). This tight cluster was found at the end of branch B.11 of *F. tularensis* subsp. *holarctica* and was divided further into two distinct genetic clades, B.45 and B.46 – each represented by multiple strains, and also a single strain (FDC310) separate from the other strains (Fig. 1b). The B.45 and B.46 clades were separated by just 12 SNPs. There were no conflicting SNP character states in the phylogeny (i.e. no homoplasy). The absence of homoplasy among 251 SNPs in the 1 531 265 nucleotide alignment of the 67 genomes added credibility to the phylogenetic reconstruction, but despite the temporal and spatial extent of our dataset, relationships among many *F. tularensis* strains within the B.45 clade remained unresolved. The clade was densely populated with 60 genomes and some of them divided into several subclades originating independently from a common internal node (Fig. 1). Such star-like phylogenetic structures with relatively long terminal branches indicate a population expansion compressed in evolutionary time. There were eight additional subclades within the B.45 clade (B.48 through B.55) that also exhibited star phylogenies. Synapomorphic SNPs shared by all of the strains within these different subclades signified their common ancestry, with 6–8 synapomorphic SNPs for the B.48 and B.52 subclades and 1–3 SNPs for the B.49–51 and B.53–55 subclades. The B.46 clade contained just six genomes and generally exhibited longer branch lengths compared with B.45, as well as a more sparsely populated hierarchical tree structure, indicating that this *F. tularensis* population was less abundant in Western Europe and had a longer evolutionary history.

### Phylogeography

**Fig. 2.**
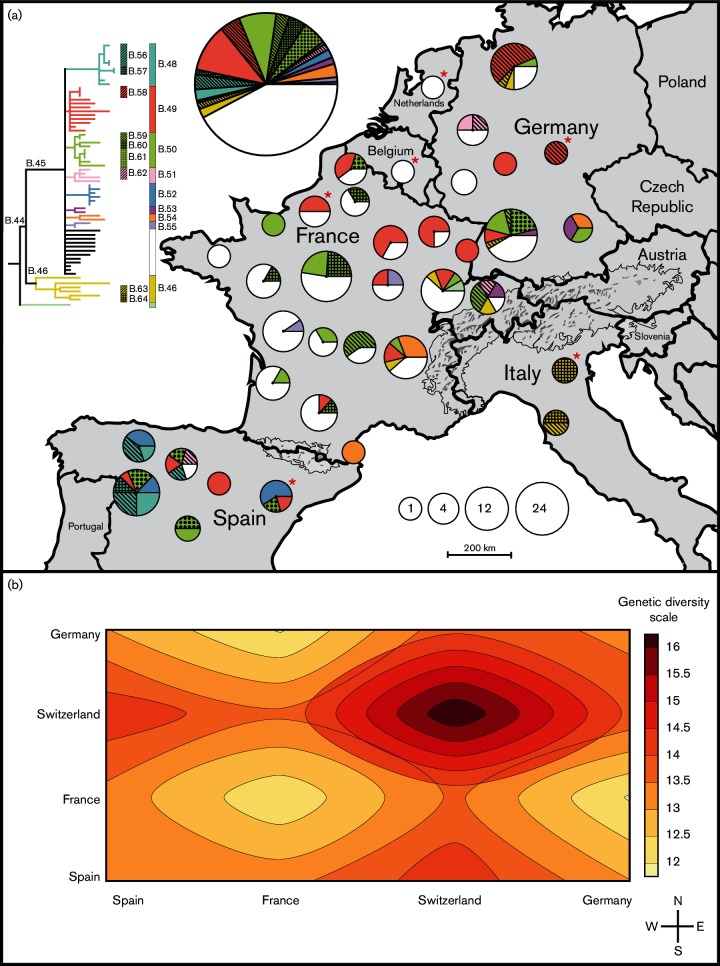
Genetic diversity and geography of *F. tularensis* in Western Europe. (a) shows phylogeographic patterns of 205 strains. The colors in the circles are consistent with the genetic clade colors of the phylogenetic tree in the upper-left. The distribution of the colors within a circle corresponds to the frequency of particular genetic clades. The size of the circle represents the number of strains isolated in the region. The large circle represents the total number of strains in the study. An asterisk indicates missing information about the exact geographical location (*n*=15). (b) shows the mean nucleotide diversity of 67 *F. tularensis* genomes among different countries ordered from West to East (x-axis) and South to North (y-axis).

Phylogeographic analyses (Fig. 2a) revealed major differences between the two main clades. Despite high sampling intensity, strains assigned to the B.46 clade (*n*=9) were isolated only towards the Eastern boundary of the study area with the majority of strains isolated in the Alps region of Switzerland and Italy; no B.46 strains were recorded west of the French Alps region. In contrast, strains assigned to clade B.45 (*n*=195) were isolated throughout Western Europe, occurring widely across the study area from east to west and from north to south. The B.45 and B.46 strains examined in this study were isolated from diverse infected hosts, with the relatively few B.46 strains being isolated from hares, humans and a lion tamarin at a zoo, indicating that under-sampling of any particular specific B.46-reservoir in Western parts of Europe is an unlikely explanation for its absence there. Several subclades within the B.45 clade were distributed throughout the study area, including subclades B.49 and B.50. However, other subclades within the B.45 clade were restricted to specific geographical locations: strains from subclades B.48 and B.52 were only isolated in Spain and strains from subclade B.54 were only isolated in the Southeast portion of the study area.

### Genetic diversity and nucleotide substitution patterns

An analysis of SNP accumulation in the 67 whole-genome sequences revealed higher genetic diversity among strains isolated in the Eastern versus the Western part of the study area. The per genome nucleotide diversity measures were different using the Jukes and Cantor correction model measuring Pi(2)JC 0.07 in the East, and Pi(2)JC 0.05 in the West. Genetic diversity was greatest among genomes from Switzerland and its neighboring countries (Fig. 2b). The SNP patterns were further explored in *F. tularensis* strains from Western Europe, aiming to infer underlying biological processes. There were 207 SNPs in predicted coding regions (145 non-synonymous and 62 synonymous) amongst the total of 251 SNPs, and we identified a prominent AT mutational bias in these genomes already containing 77.8 percent AT nucleotides. (Table 1). The total number of G or C to A or T changes was 147 and the number of A or T to G or C changes was 54. Notably, the most common changes were G→A, C→T transitions, accounting for 58 percent of the mutations in the total data, a result indicating that weak forces are acting to counteract an increase in AT-content.

**Table 1.** Number of substitutions of the six nucleotide pairs in the coding regions of the 67 genomes

Substitution	Number non-synonymous (percentage)*	Number synonymous (percentage)
C→G, G→C	0 (0)	1 (0.5)
A→C, T→G	7 (3)	1 (0.5)
A→T, T→A	12 (6)	2 (1)
C→A, G→T	21 (10)	5 (2)
A→G, T→C	24 (12)	13 (6)
G→A, C→T	81 (39)	40 (19)
Total	145	62

*Percentages were calculated as the number of the type of substitution event divided by the total of 207 substitutions, e.g. (7÷207)×100=3.

### Historical and current tularemia distribution

**Fig. 3.**
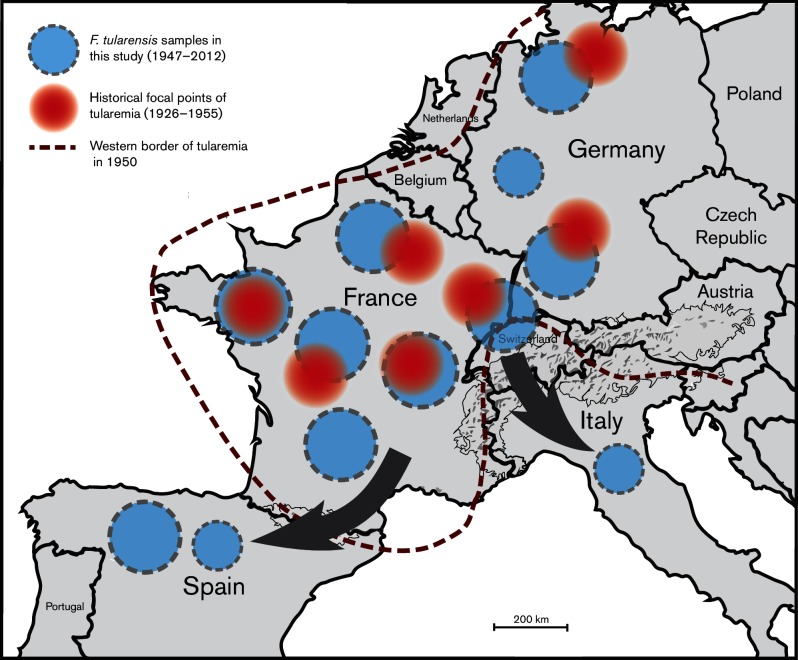
Circles in blue with dotted margins represent *F. tularensis* samples analyzed in this study (1947–2012) and the circles in red represents the historical focal points between 1926 and 1955 in the corresponding regions as reported by Jusatz *et al.* in the 1950s. The dotted red line was marked as the boundary beyond which no tularemia cases were reported between 1926 and 1950 as per Jusatz *et al*. The gray arrows show the direction of migration of *F. tularensis* in recent years.

A comparison of the geographical distribution of the strains analyzed in this study from 1947 to 2012 with historical data on tularemia epidemics from 1926–1955 (Jusatz, 1952a, b) revealed that historic disease areas largely have persisted (Fig. 3). The distribution of strains in our analysis reflected that tularemia was first reported in 1964 in Italy and 1997 in Spain indicating that these countries are new endemic areas.

### Estimate of mutation rate

Comparing the whole-genome phylogeny and the years of isolation for the corresponding *F. tularensis* isolates indicates that there was little temporal mutation signal in the dataset as a whole. Within clade B.45, with the largest number of strains whole-genome sequenced, the branch lengths are not correlated with chronological time, as strains isolated 60 years apart in Switzerland and France differed at only six SNPs even with whole-genome comparisons (Fig. 1). Using Bayesian temporal mutational analysis, our 67-genome dataset from Western Europe 1952–2012 did not have sufficient temporal structure for rate estimation. Thus, to maximize the power of the temporal mutation rate analysis, we selected whole-genome sequences representing active outbreak areas in Spain from 1998–99 (*n*=12) and 2007–08 (*n*=12) and identified a mean rate of 0.4 mutations per genome per year (1.87×10^-7^ mutations per site per year; see Fig. S1 and Table S4). The majority of mutations (*n*=45) among these 24 genomes were found at terminal tips of the phylogenetic tree and only a minority (*n*=24) were shared among multiple strains.

### Dispersal patterns

**Fig. 4.**
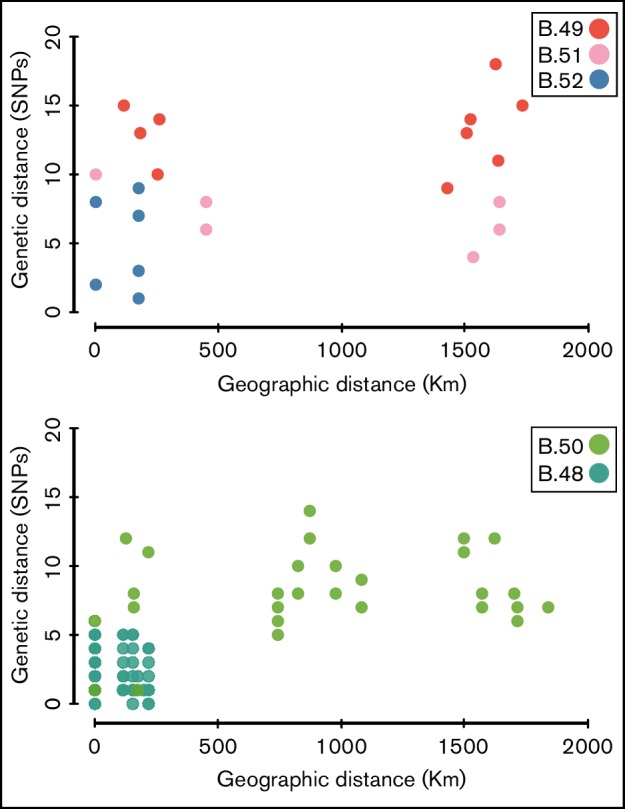
A Clade-wise comparison of genetic distance to geographic distance is plotted. The x-axis represents geographic distance between the strains in Kms and the y-axis represents genetic distance based upon SNP differences identified from whole-genome analyses. The colors of the circles are consistent with those of the clades in Fig. 1.

Overall, genetic distance correlated poorly with geographic distance among genomes within the star-like clade B.45 (Fig. 4). For example, there were small genetic distances (4–9 SNPs) between pairs of strains assigned to subclades B.49 and B.51 that were separated in geographic space by 0–1750 km. In addition, pairs of strains within subclade B.50 that were separated by very small genetic distances (≤6 SNPs) were separated in geographic space by distances ranging from 150 to over 1500 km. This pattern indicates that there are few barriers to dispersal within the study area, as very similar genomes were sometimes separated by large geographic distances. Only in the two subclades that are geographically restricted to the recently emerging areas for tularemia in Spain (subclades B.48 and B.52) was there a strong correlation between genetic distance and geographic distance.

**Fig. 5.**
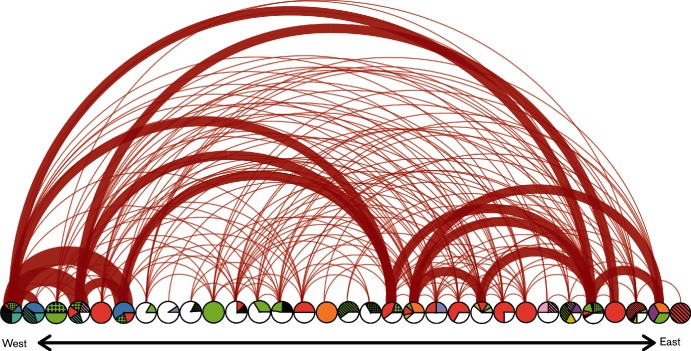
A geographical dispersal network of *F. tularensis* in continental Western Europe ordered from West to East. The pie charts at the bottom correspond to Fig. 2(a). An arc represents a possible movement of a genotype between two locations. The thickness of an arc is proportional to the number of shared genotypes at these two locations.

The genetic network analysis, which was conducted east to west across the study area using canSNP data for 205 strains, uncovered complex patterns of both local and long-distance dispersal events (Fig. 5). There were numerous examples of local dispersal events, with identical genotypes located at nearby geographic locations; this was the most abundant network pattern and was particularly common at the Eastern and Western boundaries of the study area. But there were also similar genotypes located across long geographic distances, which is consistent with past long-distance movement of recent bacterial ancestors. The network revealed many different long-distance connections between east and west locations and some intermediate-distance connections. Importantly, recent ancestors of several different genotypes appear to have been transported between the same locations (shown as thicker arcs in Fig. 5).

## Discussion

Our study confirms that Western Europe was colonized by a monophyletic population of *F. tularensis* and indicates that this primarily occurred by clonal expansion of a specific population. The founder population originated in the Eastern boundary of Western Europe, and Western and Southern regions have been colonized by clonal descendants of this founder population. Our study also demonstrates that it is possible to translate large-scale genomic microbial population data into biological properties; we found that long-range dispersal is an important feature of tularemia ecology and that *F. tularensis* mutation rates are mostly slow.

There was higher genetic diversity among *F. tularensis* samples from around the Alps, indicating a longer evolutionary history in this region as compared with other areas of Western Europe. The presence of all the major clades – B.11, B.45, B.46 and B.47 – in Switzerland but in no other area is further evidence to indicate an evolutionarily older founder population from the Eastern boundary of the study region. In contrast, there was less genetic diversity in the Western regions of the study area. We note that these findings may be consistent with a recent colonization of Western Europe starting from the East and that this would fit with epidemiology records of the first tularemia outbreaks in Southwestern areas of Spain as recently as the 1990s (Gutiérrez *et al.*, 2003; Instituto de Salud Carlos III, 1997). The star phylogeny of clade B.45 (i.e. a multi-furcation tree with many short branches connected at an internal node) contains the vast majority of all *F. tularensis* strains analyzed across Western Europe and is indicative of an evolutionary history with rapid expansion of a clone. We interpret this as a founder effect, meaning that the vast majority of *F. tularensis* in Western Europe was derived from a very small sample of an ancestral genetic pool.

Our findings support a model of *F. tularensis* biology involving outbreaks of disease being restricted to specific stationary ecosystems and landscapes, indicating that the pathogen is dependent on some specific local ecological conditions (Goethert & Telford, 2009; Svensson et al, 2009a). We found that locations of known historical tularemia outbreaks up to 1955 coincided with the distribution of strains investigated in this study, which is indicative of long-term persistence in these regions. Thus, it seems that *F. tularensis* has an ability to persist at certain locations and that this ability results in repeated outbreaks in those locations, as historically proposed by the Russian author Pavlovsky (Pavlovsky, 1966).

There was a puzzling mix of, on one hand, local genetic structure indicating micro-evolution with limited dispersal signified by identical or genetically very closely related strains from small geographic areas, and, on the other hand, clear deviations from these patterns. We found in several instances identical canSNP genotypes at distant locations, indicating that very long-distance and rapid movements of *F. tularensis* must have occurred that influenced the current genetic diversity of this bacterial population. There was also surprisingly weak correlation between genetic distance and geographic distance. We conclude that long-distance dispersal events have significantly influenced the current genetic diversity of *F. tularensis*, which would explain the observed patterns with canSNP identities at large distances (e.g. between Germany and Spain) and also the establishment of new regions of endemicity in Spain and Italy (Instituto de Salud Carlos III, 1997; Rinaldi *et al.*, 1964). Our findings support the idea that the degree of dispersal limitation may be as important for microbes as it is for plants and mammals in determining the genetic diversity of populations (Pigot & Tobias, 2015).

The mechanisms of long-range dispersal of *F. tularensis* are unknown; possibly bacteria may move rapidly by infected domestic or wild animals, or via wind (Burrows *et al.*, 2009). Infected hares may, for example, be imported from tularemia-endemic areas to previously tularemia-free areas, this has been suggested as a potential explanation for the emergence of the disease in Spain in the 1990s (Petersen & Schriefer, 2005); association with migratory birds is another possibility (Lopes de Carvalho *et al.*, 2012). The European brown hare, *Lepus europaeus*, is recognized as an important game species throughout its distribution (Smith & Johnston, 2008). The local geographical migration of this hare is described to be restricted, but conservation actions and translocations of animals may have extended the geographical range of some hare populations including in Switzerland, France, Italy and Spain (Ferretti *et al.*, 2010; Fischer & Tagand, 2012; Smith & Johnston, 2008). Wind-borne dispersal is another possible mechanism as *F. tularensis* is a prototype agent for infections acquired by inhalation of infectious aerosols (Dennis *et al.*, 2001). Large outbreaks of natural infection have repeatedly occurred via inhalation of contaminated hay or straw dust generated in farming activities (Allue *et al.*, 2008; Dahlstrand *et al.*, 1971; Johansson *et al.*, 2014; Syrjälä *et al.*, 1985). Given the well-known propensity of *F. tularensis* to be part of aerosols, and its environmental survival properties, long-distance microbial dispersal may take place over vast distances like in other microbial populations (Nguyen *et al.*, 2006; Smith *et al.*, 2013). Notably, the occurrence of long-distance transport is not equal to the successful establishment of a new *F. tularensis* outbreak area; there may be high-frequency seeding of bacteria into new geographical areas but a low chance of bacterial survival and establishment due to unsuitable ecological conditions in these new areas.

The very small genetic diversity observed among the genomes collected over a 65-year time scale and, especially, the lack of correlation between mutation accumulation and time, is remarkable. It appears that the evolutionary rate for the *F. tularensis* genetic lineages investigated here compares with, or is lower, than the lowest rates found in recent analyses of a large set of genome collections representing a range of bacterial species (Duchêne *et al.*, 2016). These results indicate that *F. tularensis* exhibits low but variable mutation rates over chronological time. We identified a mutation rate lower than one nucleotide substitution every second year per genome among a subset of strains recovered from an area in Spain; this region has emerging and recent outbreaks that should represent an area characterized by a high replication activity within the bacterial population. The overall very low or null rate of mutation in the total data set indicates a biology wherein the pathogen replicates during outbreaks and has a mechanism to survive long periods of inactivity with little replication between epidemics (Johansson *et al.*, 2014), i.e. a resting phase for long-term survival (Romanova *et al.*, 2000). Variable mutation rates related to higher replication rates during outbreaks have previously been suggested for *Yersinia pestis* (Cui *et al.*, 2013). We acknowledge that it is problematic to assess recombination within this population due to an extensive genetic homogeneity but have found no evidence to question previous conclusions of a clonal population structure (Johansson *et al.*, 2004, 2014; Larsson *et al.*, 2009). In all populations with extremely little genetic diversity it is hard to know if a SNP resulted from a *de novo* mutation or was an import by allelic exchange of a continuous DNA stretch containing this SNP. Given the lack of homoplastic SNPs in our genomic data, however, possible events of homologous recombination are unlikely to have distorted our phylogenetic tree reconstruction (Hedge & Wilson, 2014). In future studies of possible recombination in *F. tularensis,* other types of mutations like indels, tandem repeats and inversions may provide additional information.

Our observations of nucleotide substitution patterns with an extreme AT-mutation bias amongst the *F. tularensis* genomes are in agreement with the idea that ecology and lifestyle influence genetic variation (Moran *et al.*, 2008). It is likely that a recent host-adaptation of this pathogen confers strong genetic drift effects, because of repeated population bottlenecks in infected hosts, and a relaxed selection for many bacterial functions in an intracellular environment (Larsson *et al.*, 2009). The large numbers of G→A or C→T transitions and C→A or G→T transversions in the *F. tularensis* population of Western Europe signify that selective forces acting to oppose the increase in AT content indeed are weak. An alternative explanation, AT-bias because of inefficient DNA-repair systems in *F. tularensis*, seems unlikely because DNA-repair genes were found to be intact in a strain from France (Sample ID FTNF002-00 in Table S1) (Larsson *et al.*, 2009). Additional indirect evidence indicates we have captured strong genetic drift effects; the star phylogeny of the B.45 clade is probably a transient snapshot of evolution with its many subclades existing side by side in a polytomy. We have not seen such patterns in previous comparative whole-genome studies of *F. tularensis* (Afset *et al.*, 2015; Johansson *et al.*, 2014; Larsson *et al.*, 2009) and it is expected that several of these subclades will become extinct after longer evolutionary time periods, by stochastic events or because of selection forces (Kryazhimskiy & Plotkin, 2008; Rocha *et al.*, 2006; Wolf *et al.*, 2009).

In conclusion, this study demonstrates how mutation-driven microbial evolution, and particularly, a biology with a resting survival phase, genetic drift effects and long-distance geographical dispersal, have interacted to form population variation in this species. The local diversity of the tularemia pathogen is influenced by two distinct components: first, a local component containing dispersal limitation wherein bacteria are accumulating genetic diversity and expanding locally; and, second, a component of long-distance movement with a very low degree of dispersal limitation resulting in genetic diversity imports and highly similar genotypes at large distances.

## Supplementary Data

52 Supplementary File 1

## Supplementary Data

52 Supplementary File 2
