# Supplementary material for: Long-range dispersal moved Francisella tularensis into Western Europe from the East
Source: Microb Genom. 2016 Dec 12;2(12):e000100. doi: 10.1099/mgen.0.000100 (PMC5359409; doi:10.1099/mgen.0.000100)
Supplement: Supplementary File 1 [file mgen-02-100-s001.docx]

**Table S1.** Whole-genome sequences of F. tularensis samples used in this study.

| Sample ID | Alternative ID | Country, source, year | Genetic clade | Sequencing institute/ Sample provider | Illumina platform/ Read length | Total length of assembly | No. contigs | N50 | Accession number |
| --- | --- | --- | --- | --- | --- | --- | --- | --- | --- |
| Genomes made public through this work (n=62) | | | |  |  |  |  |  |  |
| F0295 | Chateauroux; FSC025 | France, hare, 1952 | B.60 | NAU^*^/FOI^†^ | GA II/102 | 1848790 | 106 | 27255 | SAMN03773721 |
| F0594 | BIM DF88 | Germany, common marmoset, 2004 | B.58 | NAU/BIM^ǂ^ | MiSeq/251 | 1853227 | 570 | 8473 | SAMN06009021 |
| F0608 | BIM DF110 | Germany, hare, 2006 | B.49 | NAU/BIM | MiSeq/251 | 1828356 | 155 | 23279 | SAMN03773724 |
| F0624 | BIM DF166 | Germany, hare, 2007 | B.63 | NAU/BIM | GA II/102 | 1863335 | 168 | 22276 | SAMN03773725 |
| F0732 | 42055/2008 | Italy, unknown, 2008 | B.64 | NAU/IZS^§^ | GA II/102 | 1854822 | 155 | 23269 | SAMN03773728 |
| F0775 | F0602; BIM DF100 | Germany, long-tailed macaque, 2002 | B.45 | NAU/BIM | MiSeq/251 | 1817209 | 99 | 27603 | SAMN03773729 |
| F0777 | F0607; BIM DF112 | Germany, common marmoset, 2006 | B.58 | NAU/BIM | MiSeq/251 | 1796685 | 100 | 27151 | SAMN03773730 |
| F0781 | F0632; BIM DF172 | Germany, hare, 2008 | B.61 | NAU/BIM | MiSeq/251 | 1801505 | 97 | 26887 | SAMN03773732 |
| F0782 | F0647; BIM DF180 | Germany, human, 2007 | B.49 | NAU/BIM | MiSeq/251 | 1861645 | 100 | 28469 | SAMN03773733 |
| F0783 | BIM F190; FT9 | Spain, unknown, 1997 | B.52 | NAU/BIM | MiSeq/251 | 1853650 | 99 | 27991 | SAMN03773734 |
| F0784 | F0638; BIM DF191 | Germany, hare, 2008 | B.45 | NAU/BIM | MiSeq/251 | 1806906 | 95 | 27538 | SAMN03773735 |
| F0799 | BIM DF233 | Germany, human, 2010 | B.49 | NAU/BIM | MiSeq/251 | 1778060 | 109 | 25220 | SAMN03773736 |
| FDC095 | CEB 02/0252; Ft "O3" | France, human ,1989 | B.49 | FOI/FOI | HiSeq 2000/100 | 1838105 | 230 | 20509 | SAMN03773837 |
| FDC099 | CEB 02/0412; Delorme | France, human, 1989 | B.54 | SciLifeLab^ǁ^/FOI | HiSeq 2000/100 | 1834543 | 424 | 12885 | SAMN03773838 |
| FDC102 | CEB 02/0415; Gorce | France, human, 1989 | B.45 | SciLifeLab/DGA^#^ | HiSeq 2000/100 | 1803707 | 1226 | 3979 | SAMN06009022 |
| FDC111 | CEB 02/0440 | France, human, 1985 | B.45 | SciLifeLab/DGA | HiSeq 2000/100 | 1842781 | 610 | 9959 | SAMN06009023 |
| FDC113 | CEB 02/0443; Gagnepain | France, human, unknown | B.54 | SciLifeLab/FOI | HiSeq 2000/100 | 1831768 | 183 | 22961 | SAMN03773839 |
| FDC206 | 2003/02318 | Belgium, hare, 2003 | B.45 | SciLifeLab/BD^**^ | HiSeq 2000/100 | 1902193 | 1080 | 6417 | SAMN06009024 |
| FDC300 | FLI 09T0179 | Germany, hare, 2009 | B.51 | FOI/FLI^††^ | MiSeq/251 | 1773021 | 150 | 21931 | SAMN03773854 |
| FDC304 | FLI JF4128 | Switzerland, human, 2008 | B.46 | FOI/FLI | MiSeq/251 | 1764636 | 244 | 15335 | SAMN03773855 |
| FDC305 | FLI JF4212 | Switzerland, human, 2008 | B.53 | FOI/FLI | MiSeq/251 | 1778068 | 127 | 24824 | SAMN03773856 |
| FDC306 | FLI JF4242 | Switzerland, hare, 2008 | B.45 | FOI/FLI | MiSeq/251 | 1825716 | 101 | 26885 | SAMN03773857 |
| FDC310 | FLI JF4429 | Switzerland, human, 2008 | B.11 | FOI/FLI | MiSeq/251 | 1804484 | 98 | 27307 | SAMN03773858 |
| FDC338 | FLI 10T0195 | Germany, hare, 2010 | B.53 | FOI/FLI | MiSeq/251 | 1789072 | 112 | 23724 | SAMN03773859 |
| FDC380 | FLI 12T0062 | Germany, human, 2012 | B.62 | FOI/FLI | MiSeq/251 | 1737112 | 448 | 6514 | SAMN06009025 |
| FDC411 | TU4 | Spain, hare,1998 | B.52 | FOI/UBU^ǂǂ^ | MiSeq/251 | 1772829 | 211 | 16136 | SAMN03773884 |
| FDC412 | TU21 | Spain, hare,1998 | B.61 | FOI/UBU | MiSeq/251 | 1778888 | 124 | 23303 | SAMN03773885 |
| FDC413 | TU39 | Spain, hare,1999 | B.48 | FOI/UBU | MiSeq/251 | 1811715 | 98 | 27307 | SAMN03773886 |
| FDC415 | TU8 | Spain, hare,1998 | B.45 | FOI/UBU | MiSeq/251 | 1827862 | 99 | 27049 | SAMN03773887 |
| FDC416 | TU17 | Spain, hare,1998 | B.61 | FOI/UBU | MiSeq/251 | 1786209 | 106 | 25587 | SAMN03773888 |
| FDC417 | TU18 | Spain, hare,1998 | B.49 | FOI/UBU | MiSeq/251 | 1781809 | 110 | 24972 | SAMN03773889 |
| FDC418 | TU16 | Spain, hare,1998 | B.52 | FOI/UBU | MiSeq/251 | 1805021 | 102 | 26885 | SAMN03773890 |
| FDC419 | TU38 | Spain, hare,1998 | B.49 | FOI/UBU | MiSeq/251 | 1785676 | 117 | 23026 | SAMN03773891 |
| FDC420 | TU23 | Spain, vole,1998 | B.48 | FOI/UBU | MiSeq/251 | 1820826 | 111 | 27307 | SAMN03773892 |
| FDC421 | TU22 | Spain, hare,1998 | B.49 | FOI/UBU | MiSeq/251 | 1776343 | 138 | 21792 | SAMN03773893 |
| FDC422 | TU31 | Spain, human,1998 | B.52 | FOI/UBU | MiSeq/251 | 1774593 | 151 | 20629 | SAMN03773894 |
| FDC423 | TU13 | Spain, hare,1998 | B.48 | FOI/UBU | MiSeq/251 | 1845697 | 98 | 27699 | SAMN03773895 |
| FDC424 | TU25 | Spain, human,1998 | B.61 | FOI/UBU | MiSeq/251 | 1846037 | 97 | 27469 | SAMN03773896 |
| FDC425 | VA-23287 | Spain, human, 2007 | B.56 | FOI/UBU | MiSeq/251 | 1776103 | 137 | 20839 | SAMN03773897 |
| FDC426 | ZA-07/2645 | Spain, human, 2008 | B.57 | FOI/UBU | MiSeq/251 | 1784006 | 108 | 25715 | SAMN03773898 |
| FDC427 | 7072002 | Spain, human, 2007 | B.56 | FOI/UBU | MiSeq/251 | 1819096 | 101 | 27306 | SAMN03773899 |
| FDC428 | 6434 | Spain, vole, 2007 | B.56 | FOI/UBU | MiSeq/251 | 1834738 | 117 | 23724 | SAMN03773900 |
| FDC429 | 3640 | Spain, hare, 2008 | B.56 | FOI/UBU | MiSeq/251 | 1850012 | 100 | 26354 | SAMN03773901 |
| FDC430 | ZA-26454 | Spain, human, 2007 | B.57 | FOI/UBU | MiSeq/251 | 1838358 | 99 | 27309 | SAMN03773902 |
| FDC431 | PA-21739 | Spain, human, 2007 | B.62 | FOI/UBU | MiSeq/251 | 1828353 | 98 | 27306 | SAMN03773903 |
| FDC432 | ZA-20206 | Spain, human, 2007 | B.56 | FOI/UBU | MiSeq/251 | 1855546 | 110 | 25748 | SAMN03773904 |
| FDC433 | 7074825 | Spain, human, 2007 | B.52 | FOI/UBU | MiSeq/251 | 1804131 | 99 | 26885 | SAMN03773905 |
| FDC434 | PA-22442 | Spain, human, 2007 | B.56 | FOI/UBU | MiSeq/251 | 1841519 | 103 | 27469 | SAMN03773906 |
| FSC026 | Charney | France, unknown, unknown | B.55 | SciLifeLab/FOI | HiSeq 2000/100 | 1856176 | 95 | 27800 | SAMN03773912 |
| FSC027 | Charantes | France, unknown, unknown | B.55 | SciLifeLab/FOI | HiSeq 2000/100 | 1850996 | 96 | 28120 | SAMN03773913 |
| FSC028 | Lat-et-Garonne | France, unknown, unknown | B.45 | SciLifeLab/FOI | HiSeq 2000/100 | 1864651 | 96 | 27838 | SAMN03773914 |
| FSC029 | St. Germaine | France, unknown, unknown | B.60 | SciLifeLab/FOI | HiSeq 2000/100 | 1852414 | 96 | 28109 | SAMN03773915 |
| FSC031 | O-407 | Italy, rabbit, 1964 | B.64 | SciLifeLab/FOI | HiSeq 2000/100 | 1858832 | 95 | 28054 | SAMN03773917 |
| FSC247 | F0020; SVA T20 | France, human, 1993 | B.45 | SciLifeLab/FOI | HiSeq 2000/100 | 1909367 | 95 | 28764 | SAMN03773988 |
| FSC455 | FT1; FTPMai-1 | Spain, hare, unknown | B.49 | SciLifeLab/FOI | GA II/39 | 1769417 | 674 | 4176 | SAMN03774110 |
| FSC456 | FT7; FTPMai-7 | Spain, human, unknown | B.61 | SciLifeLab/FOI | HiSeq 2000/100 | 1878533 | 96 | 27956 | SAMN03774111 |
| FSC555 | F0592; BIM DF1;BGA | Uncertain origin | B.63 | SciLifeLab/BIM | HiSeq 2000/100 | 1846832 | 97 | 28029 | SAMN03774138 |
| FSC599 | F0773; BIM DF76; RKI 03-01294 | Uncertain origin | B.47 | SciLifeLab/BIM | HiSeq 2000/100 | 1856942 | 96 | 28237 | SAMN03774150 |
| FT16C_B1 | None | Switzerland, castor bean tick,2012 | B.62 | SL^§§^/SL | HiSeq 2000/100 | 1805387 | 95 | 27658 | SAMN03774942 |
| FT32 | None | Switzerland, human, 2012 | B.45 | SL/SL | HiSeq 2000/100 | 1812310 | 95 | 27658 | SAMN03774932 |
| FT8C_4F | None | Switzerland, castor bean tick, 2012 | B.59 | SL/SL | HiSeq 2000/100 | 1814235 | 96 | 27658 | SAMN03774935 |
| FT9C_G7 | None | Switzerland, castor bean tick,2012 | B.59 | SL/SL | HiSeq 2000/100 | 1811355 | 96 | 27676 | SAMN03774936 |
| Genomes from continental Western Europe previously made public (n=5) | | | |  |  |  |  |  |  |
| FTNF002-00 | FTA | France, human, 2000 | B.49 | Public | NA^ǁǁ^ | 1890909 | 1 | 2E+06 | SAMN20197 |
| KO97-1026 | None | Spain (uncertain origin) | B.52 | Public | NA | 1763624 | 123 | 23683 | SAMN30633 |
| F92 | F0604; BIM; DF92; FtH_16Mai | Germany, common marmoset, 2004 | B.58 | Public | NA | 1886888 | 1 | 2E+06 | SAMN175244 |
| BD11-00177 | FDC199 | Netherlands, human, 2011 | B.45 | Public | NA | 1805001 | 96 | 27538 | SAMN177784 |
| URFT1 | None | France, human, 1997 | B.45 | Public | NA | 1761429 | 151 | 20438 | SAMN19645 |
| Additional public reference genomes used in this study (n=9) | | | |  |  |  |  |  |  |
| FSC274 | F0228 | Sweden, human, 2000 | B.10 | Public | NA | 1797320 | 99 | 25220 | ERS353713 |
| OR96-0246 | BSA | USA, 1996 | B.7 | Public | NA | 1760658 | 107 | 26683 | SAMN30669 |
| FSC844 |  | Sweden, human, 2008 | B.7 | Public | NA | 1705228 | 728 | 4171 | ERS353729 |
| FSC162 | F0162 | Sweden, human, 1995 | B.12 | Public | NA | 1887997 | 94 | 29101 | SAMN89145 |
| FSC200 | F0134 | Sweden, human | B.12 | Public | NA | 1894157 | 1 | 2E+06 | SAMN16087 |
| OSU18 | F0400 | USA, beaver, 1978 | B.4 | Public | NA | 1895727 | 1 | 2E+06 | SAMN17265 |
| MI00-1730 | F1730 | USA, human, 2000 | B.4 | Public | NA | 1765146 | 115 | 26359 | SAMN30635 |
| FSC021 | F0014; Tsuchiya | Japan, human, 1958 | B.16 | Public | NA | 1791262 | 144 | 20623 | SAMN73369 |
| FSC022 | F0015; Ebina | Japan, human, 1950 | B.16 | Public | NA | 1858313 | 90 | 28812 | SAMN19015 |

^*^NAU Northern Arizona University and Translational Genomics Research Institute (TGen), Flagstaff, USA

^†^FOI Swedish Defence Research Agency (FOI), Umeå, Sweden

^ǂ^BIM Bundeswehr Institute of Microbiology (BIM), Munich, Germany

^§^IZS National Laboratory Reference for Tularemia; Istituto Zooprofilattico Sperimentale, Italy

^ǁ^SciLifeLab Science for Life Laboratory, Uppsala, Sweden

^#^DGA DGA CBRN Defence, France

^**^BD Belgian Defence

^††^FLI Friedrich-Loeffler-Institut Federal Research Institute for Animal Health, Jena, Germany

^ǂǂ^UBU Universidad de Burgos, Burgos, Spain

^§§^SL Spiez laboratory, Spiez, Switzerland

^ǁǁ^NA Not Applicable

**Table S2.** SNP-typed *F. tularensis* samples.

| Sample ID | Alternative ID | Country, source, year | Clade | Provider |
| --- | --- | --- | --- | --- |
| F0420 | Fr001; CNEVA/2320 | France, Hare, 1996 | B.45 | ANSES^*^ |
| F0421 | Fr002; CNEVA/10178 | France, Wild boar, 1996 | B.45 | ANSES |
| F0422 | Fr003; CNEVA/11931 | France, Hare, 1996 | B.11 | ANSES |
| F0423 | Fr004; CNEVA/3 | France, Hare, 1997 | B.50 | ANSES |
| F0424 | Fr005; CNEVA/161/3469 | France, Hare, 1997 | B.45 | ANSES |
| F0425 | Fr006; CNEVA/1936 | France, Hare, 1997 | B.45 | ANSES |
| F0426 | Fr007; CNEVA/3696 | France, Hare, 1997 | B.44 | ANSES |
| F0427 | Fr008; CNEVA/4262 | France, Hare, 1997 | B.45 | ANSES |
| F0428 | Fr009; CNEVA/5838 | France, Hare, 1997 | B.45 | ANSES |
| F0429 | Fr010; CNEVA/6530 | France, Hare, 1997 | B.60 | ANSES |
| F0430 | Fr011; CNEVA/6670 | France, Hare, 1997 | B.45 | ANSES |
| F0431 | Fr012; CNEVA/12379 | France, Hare, 1997 | B.45 | ANSES |
| F0432 | Fr013; CNEVA/1086 | France, Hare, 1998 | B.45 | ANSES |
| F0434 | Fr015; CNEVA/1148 | France, Hare, 1998 | B.49 | ANSES |
| F0435 | Fr016; CNEVA/1261/2 | France ,Hare, 1998 | B.45 | ANSES |
| F0436 | Fr017; CNEVA/1386/2533 | France, Hare, 1998 | B.45 | ANSES |
| F0437 | Fr018; CNEVA/1740 | France, Hare, 1998 | B.45 | ANSES |
| F0438 | Fr019; CNEVA/1810 | France, Hare, 1998 | B.49 | ANSES |
| F0439 | Fr020; CNEVA/2115 | France, Hare, 1998 | B.45 | ANSES |
| F0440 | Fr021; CNEVA/4540 | France, Hare, 1998 | B.50 | ANSES |
| F0441 | Fr022; CNEVA/6011 | France, Hare, 1998 | B.45 | ANSES |
| F0442 | Fr023; CNEVA/7374 | France, Hare, 1998 | B.45 | ANSES |
| F0443 | Fr024; CNEVA/11263 | France, Hare, 1998 | B.45 | ANSES |
| F0444 | Fr025; CNEVA/11500 | France, Hare, 1998 | B.50 | ANSES |
| F0445 | Fr026; CNEVA/907 | France, Hare, 1999 | B.49 | ANSES |
| F0446 | Fr027; CNEVA/1927 | France, Hare, 1999 | B.45 | ANSES |
| F0447 | Fr028; AFSSA/2772 | France, Hare, 1999 | B.45 | ANSES |
| F0448 | Fr029; AFSSA/4071 | France, Hare, 1999 | B.45 | ANSES |
| F0449 | Fr030; AFSSA/4613 | France, Hare, 1999 | B.60 | ANSES |
| F0450 | Fr031; AFSSA/4949 | France, Hare, 1999 | B.49 | ANSES |
| F0451 | Fr032; AFSSA/5398 | France, Hare, 1999 | B.45 | ANSES |
| F0452 | Fr033; AFSSA/5464 | France,Hare,1999 | B.45 | ANSES |
| F0454 | Fr035; AFSSA/6442/10591 | France, Hare, 1999 | B.54 | ANSES |
| F0455 | Fr036; AFSSA/6446 | France, Hare, 1999 | B.45 | ANSES |
| F0456 | Fr037; AFSSA/10514 | France, Hare, 1999 | B.45 | ANSES |
| F0457 | Fr038; AFSSA/11139 | France, Hare, 1999 | B.60 | ANSES |
| F0458 | Fr039; AFSSA/11155 | France, Hare, 1999 | B.61 | ANSES |
| F0460 | Fr041; AFSSA/859 | France, Hare, 2000 | B.50 | ANSES |
| F0461 | Fr042; AFSSA/946 | France, Hare, 2000 | B.45 | ANSES |
| F0462 | Fr043; AFSSA/1296 | France, Hare, 2000 | B.45 | ANSES |
| F0463 | Fr044; AFSSA/1829 | France, Hare, 2000 | B.45 | ANSES |
| F0464 | Fr045; AFSSA/2748 | France, Hare, 2000 | B.45 | ANSES |
| F0465 | Fr046; AFSSA/2899 | France, Hare, 2000 | B.45 | ANSES |
| F0466 | Fr047; AFSSA/3244 | France, Hare, 2000 | B.61 | ANSES |
| F0467 | Fr048; AFSSA/3423/6 | France, Hare, 2000 | B.49 | ANSES |
| F0468 | Fr049; AFSSA/3578 | France, Hare, 2000 | B.45 | ANSES |
| F0469 | Fr050; AFSSA/8403 | France, Hare, 2000 | B.50 | ANSES |
| F0470 | Fr051; AFSSA/9955 | France, Hare, 2000 | B.45 | ANSES |
| F0471 | Fr052; AFSSA/197 | France, Hare, 2001 | B.49 | ANSES |
| F0472 | Fr053; AFSSA/1710 | France, Hare, 2001 | B.45 | ANSES |
| F0473 | Fr054; AFSSA/3098 | France, Hare, 2001 | B.45 | ANSES |
| F0474 | Fr055; AFSSA/4620 | France, Hare, 2001 | B.45 | ANSES |
| F0475 | Fr056; AFSSA/5603 | France, Hare, 2001 | B.45 | ANSES |
| F0476 | Fr057; AFSSA/6351 | France, Hare, 2001 | B.45 | ANSES |
| F0477 | Fr058; AFSSA/9180 | France, Hare, 2001 | B.45 | ANSES |
| F0478 | Fr059; AFSSA/9261/2 | France, Hare, 2001 | B.49 | ANSES |
| F0479 | Fr060; AFSSA/274 | France, Hare, 2002 | B.45 | ANSES |
| F0480 | Fr061; AFSSA/2023 | France, Hare, 2002 | B.54 | ANSES |
| F0481 | Fr062; AFSSA/2528 | France, Hare, 2002 | B.45 | ANSES |
| F0482 | Fr063; AFSSA/3449 | France, Hare, 2002 | B.45 | ANSES |
| F0483 | Fr064; AFSSA/3611 | France, Hare, 2002 | B.49 | ANSES |
| F0484 | Fr065; AFSSA/3820 | France, Hare, 2002 | B.45 | ANSES |
| F0485 | Fr066; AFSSA/5143 | France, Primate, 2002 | B.45 | ANSES |
| F0486 | Fr067; AFSSA/5452 | France, Hare, 2002 | B.45 | ANSES |
| F0487 | Fr068; AFSSA/6821 | France, Hare, 2002 | B.45 | ANSES |
| F0488 | Fr069; AFSSA/7124 | France, Human, 2002 | B.45 | ANSES |
| F0489 | Fr070; AFSSA/8108 | France, Hare, 2002 | B.49 | ANSES |
| F0490 | Fr071; AFSSA/97 | France, Hare, 2003 | B.50 | ANSES |
| F0491 | Fr072; AFSSA/292 | France, Hare, 2003 | B.45 | ANSES |
| F0492 | Fr073; AFSSA/571 | France, Hare, 2003 | B.45 | ANSES |
| F0493 | Fr074; AFSSA/782 | France, Human, 2003 | B.49 | ANSES |
| F0494 | Fr075; AFSSA/824 | France, Hare, 2003 | B.54 | ANSES |
| F0495 | Fr076; AFSSA/1207 | France, Hare, 2003 | B.61 | ANSES |
| F0496 | Fr077; AFSSA/1458 | France, Hare, 2003 | B.61 | ANSES |
| F0497 | Fr078; AFSSA/1563 | France, Hare, 2003 | B.61 | ANSES |
| F0498 | Fr079; AFSSA/1685 | France, Hare, 2003 | B.45 | ANSES |
| F0499 | Fr080; AFSSA/2698 | France, Hare, 2003 | B.49 | ANSES |
| F0500 | Fr081; AFSSA/4039 | France, Hare, 2003 | B.49 | ANSES |
| F0501 | Fr082; AFSSA/4640 | France, Hare, 2003 | B.49 | ANSES |
| F0502 | Fr083; AFSSA/4674 | France, Hare, 2003 | B.61 | ANSES |
| F0503 | Fr084; AFSSA/5120 | France, Hare, 2003 | B.49 | ANSES |
| F0504 | Fr085; AFSSA/5220 | France, Hare, 2003 | B.45 | ANSES |
| F0505 | Fr086; AFSSA/5566 | France, Hare, 2003 | B.60 | ANSES |
| F0506 | Fr087; AFSSA/5905 | France, Hare, 2003 | B.45 | ANSES |
| F0507 | Fr088; AFSSA/48 | France, Hare, 2004 | B.45 | ANSES |
| F0508 | Fr089; AFSSA/250 | France, Hare, 2004 | B.45 | ANSES |
| F0509 | Fr090; AFSSA/293 | France, Human, 2004 | B.60 | ANSES |
| F0510 | Fr091; AFSSA/574 | France, Hare, 2004 | B.60 | ANSES |
| F0511 | Fr092; AFSSA/532 | France, Hare, 2004 | B.45 | ANSES |
| F0512 | Fr093; AFSSA/1011 | France, Hare, 2004 | B.60 | ANSES |
| F0513 | Fr094; AFSSA/1168 | France, Hare, 2004 | B.50 | ANSES |
| F0514 | Fr095; AFSSA/1165 | France, Hare, 2004 | B.45 | ANSES |
| F0515 | Fr096; AFSSA/1421 | France, Hare, 2004 | B.45 | ANSES |
| F0516 | Fr097; AFSSA/3814 | France, Human, 2004 | B.45 | ANSES |
| F0517 | Fr098; AFSSA/71 | France, Human, 2004 or 2005 | B.50 | ANSES |
| F0518 | Fr099; AFSSA/177 | France, Hare, 2004 or 2005 | B.49 | ANSES |
| F0519 | Fr100; AFSSA/841 | France, Hare, 2004 or 2005 | B.45 | ANSES |
| F0520 | Fr101; AFSSA/842 | France, Hare, 2004 or 2005 | B.45 | ANSES |
| F0521 | Fr102; Réf.CIP/242 | France, Hare, 1947 | B.45 | ANSES |
| F0522 | Fr103; Réf.CIP/243 | France, Hare, 1947 | B.45 | ANSES |
| F0593 | BIM DF89 | Germany, Common marmoset , 2004 | B.58 | BIM^†^ |
| F0600 | BIM CHF102 | Switzerland, Lion Tamarin, 2002 | B.46 | BIM |
| F0605 | BIM DF91 | Germany, Common marmoset, 2004 | B.58 | BIM |
| F0606 | BIM DF90 | Germany, Common marmoset, 2004 | B.58 | BIM |
| F0609 | BIM DF109 | Germany, Long-tailed macaque, 2005 | B.45 | BIM |
| F0610 | BIM DF108 | Germany, Long-tailed macaque, 2005 | B.45 | BIM |
| F0612 | BIM DF106 | Germany, Vole, 2005 | B.58 | BIM |
| F0613 | BIM DF105 | Germany, Vole, 2005 | B.58 | BIM |
| F0618 | BIM DF157 | Germany, Hare, 2007 | B.61 | BIM |
| F0619 | BIM DF156 | Germany, Hare, 2007 | B.45 | BIM |
| F0622 | BIM DF168 | Germany, Hare, 2007 | B.45 | BIM |
| F0623 | BIM DF167 | Germany, Hare, 2007 | B.61 | BIM |
| F0635 | BIM DF199 | Germany, Hare, 2008 | B.54 | BIM |
| F0644 | BIM DF177 | Germany, Hare, 2008 | B.45 | BIM |
| F0645 | BIM DF178 | Germany, Hare, 2008 | B.61 | BIM |
| F0648 | BIM DF181 | Germany, Human, 2008 | B.45 | BIM |
| F0733 | 21851/2006 | Italy, Hare, 2006 | B.63 | IZS^ǂ^ |
| F0734 | 5768/2001 | Italy, Human, 2001 | B.64 | IZS |
| F0786 | BIM DF212 | Germany, Hare, unknown | B.45 | BIM |
| F0787 | BIM DF216 | Germany, Unknown, Unknown | B.45 | BIM |
| F0788 | BIM DF218 | Germany, Hare, 2009 | B.61 | BIM |
| F0790 | BIM DF221 | Germany, Human, 2010 | B.45 | BIM |
| F0791 | BIM DF223 | Germany, Hare, 2010 | B.61 | BIM |
| F0792 | BIM DF226 | Germany, Human, 2010 | B.61 | BIM |
| F0793 | BIM DF230 | Germany, Hare, 2010 | B.45 | BIM |
| F0794 | BIM DF231 | Germany, Unknown, unknown | B.61 | BIM |
| F0796 | BIM DF235 | Germany, Human, unknown | B.53 | BIM |
| F0797 | BIM DF237 | Germany, Hare, 2010 | B.45 | BIM |
| F0798 | BIM DF239 | Germany, Hare, 2010 | B.11 | BIM |
| F0284 | SP98-2108 | Spain, Human, 1998 | B.52 | CDC^§^ |
| F0326 | SP98-6120 | Spain, Human, 1998 | B.61 | CDC |
| F0871 | 3597B | Spain, Hare, 2004 | B.48 | ISC III^ǁ^ |
| F0872 | FT7 | Spain, Human, 1998 | B.50 | ISC III |
| F0422 | Fr003; CNEVA/11931 | France, Hare, 1996 | B.11 | ANSES |
| F0453 | Fr034; AFSSA/5719 | France, Hare, 1999 | B.45 | ANSES |
| FDC414 | TU27 | Spain, Human, 1998 | B.48 | ITA^#^ |
| F0874 | FT33 | Spain, Unknown, 1998 | B.48 | ISC III |
| F0878 | BZO18 | Spain, Human, 2007 | B.56 | ISC III |

^*^ANSES French Agency for Food, Environmental and Occupational Health & Safety

^†^BIM Bundeswehr Institute of Microbiology (BIM), Munich, Germany

^ǂ^IZS National Laboratory Reference for Tularemia; Istituto Zooprofilattico Sperimentale

^§^CDC Center for Disease Control and Prevention, USA

^ǁ^ISC III Instituto de Salud Carlos III, Majadahonda, Madrid, Spain

^#^ITA Intituto Tecnológico Agrario, Consejería de Agricultura y Ganadería, Spain

**Table S3.** CanSNP assays developed for this study.

| SNP | SCHU S4 position | FSC200 position | LVS position | Genome SNP state (D/A)^*^ | Melt MAMA primer^†^ | Melt-MAMA primer sequence^ǂ^ | Primer concentration (0.2 µM) | Annealing temperature (°C) |
| --- | --- | --- | --- | --- | --- | --- | --- | --- |
| B.44 | 425,910 | 469,203 | 467,254 | C/T | A | CTATGAGGCTATAGATACTGCGgT | 1:1 | 60 |
|  |  |  |  |  | D | ggggcggggcggggcATGACTATGAGGCTATAGATACTGCtAC |  |  |
|  |  |  |  |  | C | ACCTTTCTCTTAAAGAGTCTATAATAGAGTT |  |  |
| B.45 | 810,993 | 1,361,832 | 1,359,860 | A/G | A | GTGTTACCTTCGAATATAAGAgCG | 1:1 | 55 |
|  |  |  |  |  | D | ggggcggggcggggcGGTGTTACCTTCGAATATAAGAtCA |  |  |
|  |  |  |  |  | C | GCTTCAAGTAAAACTAACCCAAAA |  |  |
| B.46 | 972,159 | 1,191,222 | 1,189,252 | A/G | A | CAAAAGCTTGCTTATACTCcGC | 4:1 | 60 |
|  |  |  |  |  | D | ggggcggggcggggcTCAAAAGCTTGCTTATACTCgGT | 4Anc:1Der |  |
|  |  |  |  |  | C | AATTGACTATTCAGCATTTGTTAAAGAA |  |  |
| B.47 | 334,426 | 231,074 | 231,131 | C/T | A | CCAATATAGCTTGTCACTTCAAATgTA | 1:1 | 55 |
|  |  |  |  |  | D | ggggcggggcggggcAATATAGCTTGTCACTTCAAATCgG |  |  |
|  |  |  |  |  | C | TTAGAAAAGTGGCTCGTGTAAGATT |  |  |
| B.48 | 45,178 | 1,748,783 | 1,750,621 | A/G | A | CACTATGACATAGAAGCTTGCtGC | 1:1 | 63 |
|  |  |  |  |  | D | ggggcggggcggggcTACACTATGACATAGAAGCTTGCCaT |  |  |
|  |  |  |  |  | C | CTATTGGCAACAACATTAAATCCTC |  |  |
| B.49 | 328,029 | 224,673 | 224,730 | A/G | A | ATTCAGCAAAATTTAAAATATCCTGtG | 1:4 | 60 |
|  |  |  |  |  | D | ggggcggggcggggcCAGCAAAATTTAAAATATCCTGgA | 1Anc:4Der |  |
|  |  |  |  |  | C | AATATTATTTTAACTAGAAACAAAGATTACAAAC |  |  |
| B.50 | 1,045,578 | 1,010,080 | 1,008,109 | A/C | A | GAGAAAGATAGTTACCGAGGcG | 1:1 | 60 |
|  |  |  |  |  | D | ggggcggggcggggcACTGAGAAAGATAGTTACCGAGcAT |  |  |
|  |  |  |  |  | C | TATTCCTTTACCTTTTAAGATAGTACTCCA |  |  |
| B.51 | 1,317,925 | 412,149 | 410,201 | A/G | A | GTGAGATAGATCTATAGCTATTCAAATGcC | 1:2 or 1:4 | 55 |
|  |  |  |  |  | D | ggggcggggcggggcGTGAGATAGATCTATAGCTATTCAAATGaT | 1Anc:2Der or 1Anc:4Der |  |
|  |  |  |  |  | C | CATAATTGATTATACTTATTTCTATTTTCG |  |  |
| B.52 | 403,372 | 444,660 | 442,711 | T/C | A | CTACAGGAAAAACCGCAAAtC | 5:1 | 60 |
|  |  |  |  |  | D | ggggcggggcggggcCTACAGGAAAAACCGCAAAaT | 5Anc:1Der |  |
|  |  |  |  |  | C | GCAACAAATAACACTTGGGGAG |  |  |
| B.53 | 1,496,284 | 600,619 | 598,669 | A/C | A | GTTACTGGTAGTCATATTCCAGAcG | 4:1 | 60 |
|  |  |  |  |  | D | ggggcggggcggggcTTACTGGTAGTCATATTCCAGcGT | 4Anc:1Der |  |
|  |  |  |  |  | C | TATGGAGTATTAAACTTAATACCATTTCTAT |  |  |
| B.54 | 76,601 | 1,716,393 | 1,718,231 | T/C | A | CCTTCACGACAAGATCTTCTTACAG | 4:1 | 55 |
|  |  |  |  |  | D | ggggcggggcggggcCCTTCACGACAAGATCTTCTTAGAA | 4Anc:1Der |  |
|  |  |  |  |  | C | CTCTAGAATTAATTAAAGAGCAGGATC |  |  |
| B.55^§^ | 849,387 | 311,788 | 311,845 | C/T | A | TATCTTGGACTCCAAAAACTATATAAcT | 1:1 | 60 |
|  |  |  |  |  | D | ggggcggggcggggcTATCTTGGACTCCAAAAACTATATAAtC |  |  |
|  |  |  |  |  | C | TTTTACAAAACTATCTGATGCGAT |  |  |
| B.56^§^ | 1,129,504 | 828,210 | 826,239 | T/C | A | ACCAGTTTGCACATAGTGTAGTCAaG | ^ǁ^Use in non-competitive form | 55 |
|  |  |  |  |  | D | ggggcggggcggggcACCAGTTTGCACATAGTGTAGTCaTA |  |  |
|  |  |  |  |  | C | CTTTTAAAAAACAAAAATCCAAGAA |  |  |
| B.57 | 226,892 | 1,864,164 | 1,866,001 | A/G | A | ATAGTTTCGGTGATACCAACTACaG | 4:1 | 60 |
|  |  |  |  |  | D | ggggcggggcggggcCATAGTTTCGGTGATACCAACTAaTA | 4Anc:1Der |  |
|  |  |  |  |  | C | CAAACAAGTCACAGATAATGTTACAAG |  |  |
| B.58 | 619,228 | 847,774 | 845,803 | A/G | A | CTGGTCTAGAGATTGGTGACgG | 4:1 | 55 |
|  |  |  |  |  | D | ggggcggggcggggcAGTAACTGGTCTAGAGATTGGTGAgTA | 4Anc:1Der |  |
|  |  |  |  |  | C | ACCAGGTATTAAGGCTGTTAGAAAC |  |  |
| B.59 | 1,457,411 | 640,613 | 638,663 | A/G | A | AATATCTCTACTACAGTTAAATCTCAAGAaTC | ^ǁ^Use in non- | 60 |
|  |  |  |  |  | D | ggggcggggcggggcAATATCTCTACTACAGTTAAATCTCAAGAgTT | competitive |  |
|  |  |  |  |  | C | CAACAATAGCGCTTAAAAGCTTC | form |  |
| B.60 | 366,403 | 1,242,822 | 1,240,852 | A/G | A | TTGATAAGTTCAAGAAGAGATTTGGtG | 4:1 | 60 |
|  |  |  |  |  | D | cgggcgggcgggcgggTTGATAAGTTCAAGAAGAGATTTGGaA | 4Anc:1Der |  |
|  |  |  |  |  | C | GCTGGTTTAGTTTGCTGCGA |  |  |
| B.61 | 339,130 | 235,778 | 235,835 | A/G | A | AACCTTAACTTCAAATAGCTTCTCTaC | 4:1 | 65 |
|  |  |  |  |  | D | cgggcgggcgggcgggAACCTTAACTTCAAATAGCTTCTCTgT | 4anc:1Der |  |
|  |  |  |  |  | C | TTCAACTATAGTATTCGAGGTGGCT |  |  |
| B.62 | 1,669,333 | 505,691 | 503,742 | T/C | A | GTTAAATAGAGCTAAAAAAACAAATTTGGTC | 4:1 | 60 |
|  |  |  |  |  | D | ggggcggggcggggcGTTAAATAGAGCTAAAAAACAAAATTTGcTT | 4anc:1Der |  |
|  |  |  |  |  | C | TACTTGTTCGCCACGTAATACCG |  |  |
| B.63 | 959,431 | 1,204,782 | 1,202,812 | A/G | A | ATCTTACCATATTTAAATCTGCTAATAGAGTAtAG | 4:1 | 55 |
|  |  |  |  |  | D | ggggcggggcggggcATCTTACCATATTTAAATCTGCTAATAGAGTAGtA | 4Anc:1Der |  |
|  |  |  |  |  | C | TAGCAAGTATTATGCTTGTCATATTGATG |  |  |
| B.64 | 974,259 | 1,189 903 | 1,187,933 | T/A | A | ACGATAGAGTTTCAATTTTTACTGCTGATgTT | 4:1 | 60 |
|  |  |  |  |  | D | ggggcggggcggggcCGATAGAGTTTCAATTTTTACTGCTGATTcA | 4anc:1Der |  |
|  |  |  |  |  | C | TGATTTTGCTAAAGCAATAAAACAATCACTA |  |  |

^*^SNP states are presented according to their orientation in the SCHU S4 reference genome (NC_006570); D: Derived SNP state; A: Ancestral SNP state.

^†^Melt-mismatch amplification mutation assay (MAMA), D: Derived; A: Ancestral; C: Common.

^ǂ^Primer tails and antepenultimate mismatch bases are in lower case.

^§^This assay should be considered partially validated. Tested on synthetic templates only.

^ǁ^Use the two Forward allele-specific primers in separate reactions to test as non-competitive reactions rather than combining them for a competitive reaction.

**Table S4.** Mean rate and parameters of the mutation rate analysis using BEAST.

| Parameter | Value |
| --- | --- |
| Mean | 1,8712E-7 |
| Std err of mean | 1,2736E-9 |
| Median | 1,7572E-7 |
| Geometric mean | 1,6957E-7 |
| 95% HPD lower^*^ | 4,8783E-8 |
| 95% HPD upper | 3,5254E-7 |
| Auto-correlation time (ACT) | 22484,1381 |
| Effective sample size (ESS) | 4003,2666 |

^*^The 95% HPD stands for 95% Highest Posterior Density interval, upper and lower bounds.
