# Supplementary material for: Long-range dispersal moved Francisella tularensis into Western Europe from the East
Source: Microb Genom. 2016 Dec 12;2(12):e000100. doi: 10.1099/mgen.0.000100 (PMC5359409; doi:10.1099/mgen.0.000100)

Fig. S1. Geographic distribution of the outbreak strains from Spain (a) and a display of the mutation rate estimate using the BEAST software (b).


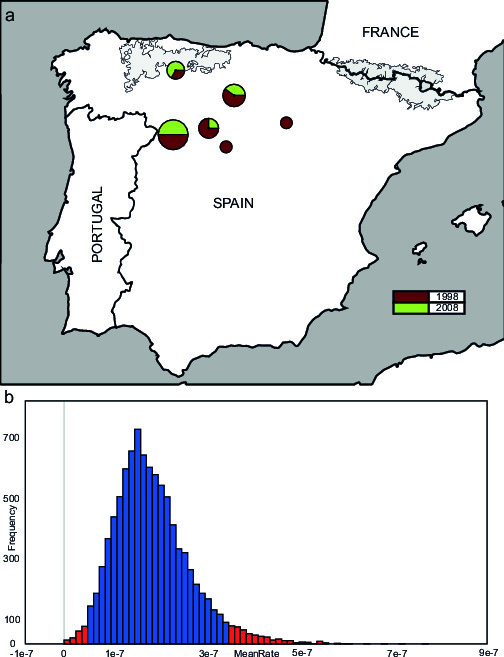

Supplement: Supplementary File 2 [file mgen-02-100-s002.docx]
